# Innate Sleep Apnea in Spontaneously Hypertensive Rats Is Associated With Microvascular Rarefaction and Neuronal Loss in the preBötzinger Complex

**DOI:** 10.1161/STROKEAHA.123.044732

**Published:** 2023-11-28

**Authors:** Reno Roberts, Robert T.R. Huckstepp

**Affiliations:** School of Life Sciences, University of Warwick, Coventry, United Kingdom.

**Keywords:** cognition, hypertension, sleep apnea syndromes

## Abstract

**BACKGROUND:**

Sleep apnea (SA) is a major threat to physical health and carries a significant economic burden. These impacts are worsened by its interaction with, and induction of, its comorbidities. SA holds a bidirectional relationship with hypertension, which drives atherosclerosis/arteriolosclerosis, ultimately culminating in vascular dementia.

**METHODS:**

To enable a better understanding of these sequelae of events, we investigated innate SA and its effects on cognition in adult-aged spontaneously hypertensive rats, which have a range of cardiovascular disorders: plethysmography and electroencephalographic/electromyographic recordings were used to assess sleep-wake state, breathing parameters, and sleep-disordered breathing; immunocytochemistry was used to assess vascular and neural health; the forced alteration Y maze and Barnes maze were used to assess short- and long-term memories, respectively; and an anesthetized preparation was used to assess baroreflex sensitivity.

**RESULTS:**

Spontaneously hypertensive rats displayed a higher degree of sleep-disordered breathing, which emanates from poor vascular health leading to a loss of preBötzinger Complex neurons. These rats also display small vessel white matter disease, a form of vascular dementia, which may be exacerbated by the SA-induced neuroinflammation in the hippocampus to worsen the related deficits in both long- and short-term memories.

**CONCLUSIONS:**

Therefore, we postulate that hypertension induces SA through vascular damage in the respiratory column, culminating in neuronal loss in the inspiratory oscillator. This induction of SA, which, in turn, will independently exacerbate hypertension and neural inflammation, increases the rate of vascular dementia.

Sleep apnea (SA) is estimated to affect 1.5 million UK adults, equivalent to ≈4% of men and ≈2% of women. However, up to 85% of cases are undiagnosed,^1^ meaning that prevalence is closer to ≈50% of men and ≈25% of women.^2^ This rise may be in part due to factors such as obesity and hypertension,^3,4^ which are steadily rising in developing countries.^5^

Dyspneic episodes result in temporary but immediate elevations in blood pressure. The associated blood oxygen desaturation coupled with prolonged sympathetic activation may cause sustained hypertension.^6^ As greater rates of dyspneic episodes lead to hypertension^6^ and increasing severity of SA is associated with linear rises in blood pressure,^7^ SA has long been considered a risk factor for hypertension, though the association between hypertension and SA may be age dependent.^8^

Hypertension is a major risk factor for the world’s most prevalent killers, including coronary heart disease and stroke.^9^ Moreover, hypertension^10^ and SA^11^ are leading causes of vascular dementia (VaD). The resulting release of proinflammatory cytokines caused by intermittent hypoxic hypercapnia during SA undoubtedly increases structural damage to endothelial cells of blood vessels in the brain,^12^ a contributing factor to VaD. Vascular cognitive impairment, a complication of cerebrovascular disease,^13^ involves a wide range of disorders, the most severe being VaD. These cerebrovascular pathologies are more widespread in neurodegenerative diseases with cognitive decline, most of which are linked directly to hypertension as a risk factor for developing the disease in midlife.^14^

Breathing is controlled by the respiratory microcircuit, which includes the central pattern generator for inspiratory rhythm generation, the preBötzinger Complex (preBötC).^15^ Disruption of the preBötC can lead to a complete loss of respiration, ataxic breathing, or SA.^16,17^ SA induced through loss of preBötC neurons induces hippocampal neuroinflammation and deficits in short- and long-term memories.^17^ The interplay between SA and VaD may be exacerbated by the combination of vascular damage and neuroinflammation.

Spontaneously hypertensive rats (SHRs) display cognitive deficits due to small vessel white matter disease.^18^ SHRs develop cardiovascular pathologies that correlate with the progression of hypertension.^19^ SHRs also display SA, which can be blocked by normalizing blood pressure.^20^ While hypertension reduces airway patency in a way that could induce SA,^21^ we think that other mechanisms may contribute. We postulate that microvascular rarefaction in the preBötC of SHRs leads to cell death and ultimately SA. SA then drives, or exacerbates, hypertension, microvascular rarefaction, and neural inflammation to cause cognitive decline and eventually dementia. Given the relationship between SA and hypertension, this will drive a downward spiral amplifying VaD in these rats.

## METHODS

Brief methods are given. Full detailed methods are available in the Supplemental Material; this article follows the ARRIVE (Animal Research: Reporting of In Vivo Experiments) reporting guideline.^22^

### Data Availability

The authors confirm that the data supporting the findings of this study are available in the article and are available from the corresponding author upon request.

### Ethics

All experiments involving animals were approved by the University of Warwick Animal Welfare and Ethical Review Board in accordance with the UK Animals (Scientific Procedures) Act (1986) and the EU Directive 2010/63/EU and performed under a project license issued by the UK Home Office.

### Animals

Adult (6–8 months old) male Wistar-Kyoto (WKY) rats (417±35 g; n=9) and SHRs (406±54 g; n=8) were used.

### Sleep and Breathing Recordings

Four electroencephalographic electrodes were inserted into the brain, and 2 electromyographic wires were inserted into the trapezius muscle. Animals were allowed to recover 2 weeks postoperative. Rats were placed into a plethysmography chamber for a single 3-hour recording per week for 7 weeks during the light phase of the light/dark cycle. Airflow recorded via a pressure transducer connected to the plethysmography chamber was used to calculate breathing parameters. Electroencephalographic/electromyographic signals were used to assign sleep-wake state. All sleep states and apnea-hypopnea index count analysis are expressed as per hour of sleep for consistency across the recordings, and sigh counts are expressed as per combined hours of sleep and quiet wakefulness.

### Cognitive Tests

Following the assessment of SA severity, rats underwent cognitive tests. The Y maze (forced alteration) was tested twice; the first with 1 arm blocked (10 minutes) and the second with both arms open (5 minutes) on the same day with 1 hour between sessions. The Barne maze was tested in 10-minute sessions for 12 consecutive days.

### Baroreflex Sensitivity Test

Rats were anesthetized and maintained throughout surgery with urethane (1 g/kg) and α-chloralose (50 mg/kg). Additional doses of α-chloralose were administered as required. Body temperature was maintained at 36.5 °C. The femoral artery was connected to pressure transducers to record blood pressure. Phenylephrine (3.5 mg/mL) or sodium nitroprusside (35 mg/mL) was administered to test baroreflex sensitivity. Data are displayed as the ratio of the mean arterial pressure (MAP) or heart rate after injection compared with the average baseline of each animal.

### Tissue Collection

At the end of the baroreflex sensitivity tests, rats were transcardially perfused with 4% paraformaldehyde, fixed overnight at 4 °C, and then stored in cryoprotectant (30% sucrose+0.02% sodium azide) at 4 °C.

### Immunocytochemistry

Tissue was sectioned to 50 µm on a cryostat before undergoing heat-activated antigen retrieval. Free-floating slices were blocked with 5% BSA for 1 hour at room temperature. Slices were then incubated overnight at room temperature in primary antibodies with BSA and 0.1% triton.

Medulla: mouse anti-NeuN (neuronal nuclear antigen; 1:100, MAB337) and rabbit anti-NK1R (neurokinin-1 receptor; 1:500, ab5060).
Microvascular rarefaction: rabbit anti-VCAM (vascular cell adhesion molecule; 1:200, ab134047), mouse anti-Tie2/TEK (angiopoietin-1 receptor; 1:200, ab33), and goat anti-ChAT ([choline acetyl transferase]; 1:50, ab144P).
Neuroinflammation: goat anti-Iba-1 (ionized calcium-binding adaptor molecule 1; 1:83, ab5076).

The slices were washed and placed in a blocking solution for 1 hour at room temperature. Slices were then incubated for 2 hours at room temperature with secondary antibodies with BSA and 0.1% triton.

Medulla: donkey anti-mouse Alexa Fluor 568 (1:250, A10037) and donkey anti-rabbit Alexa Fluor 488 (1:250, A10037).
Microvascular rarefaction: donkey anti-rabbit Alexa Fluor 568 (1:250, ab150074), donkey anti-mouse Alexa Fluor 488 (1:250, A-21202), and donkey anti-goat Alexa Fluor 405 (1:250, ab175665).
Neuroinflammation: donkey anti-goat Alexa Fluor 488 (1:250, A10037) and DAPI (4′,6-diamidino-2-phenylindole) staining solution (1:1000, ab228549).

Slices were mounted on polylysine microscope slides, dehydrated overnight, rehydrated, and coverslipped. Slides were imaged on a Zeiss 880 confocal microscope. All image processing and analyses were performed on Zen software followed by ImageJ.

### Data Analysis

All experimental units are a rat, and all technical repeats are averaged to create a biological repeat for analysis. Outliers were removed, and data were tested for normality using Shapiro-Wilks tests before statistical tests were performed in OriginPro.

## RESULTS

### SHRs Are Innately Hypertensive

**Figure 1.**
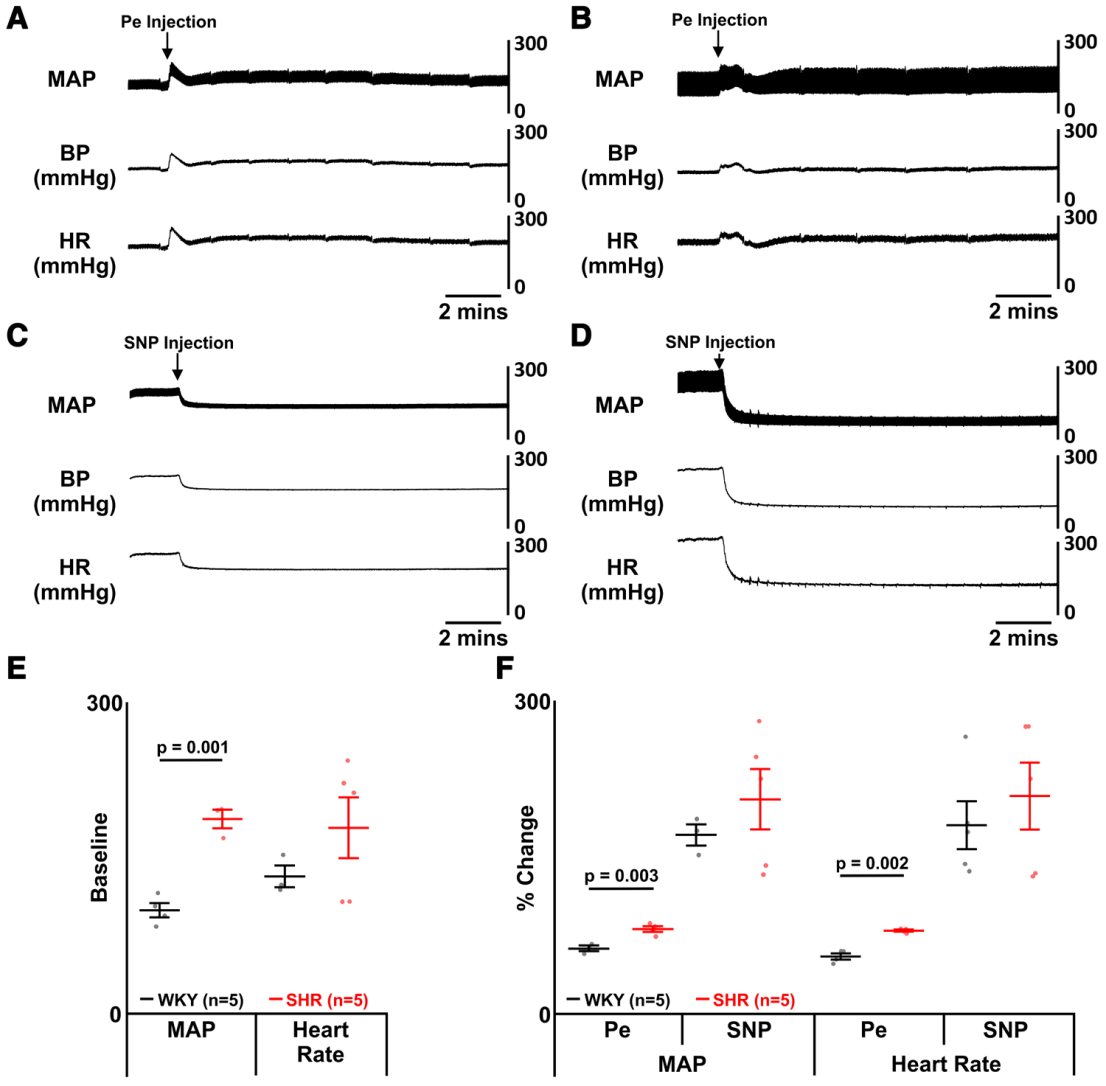
**Spontaneously hypertensive rats (SHRs) have higher blood pressure and altered baroreflex sensitivity. A**, Representative trace showing mean arterial pressure (MAP), blood pressure (BP), and heart rate (HR) in a Wistar-Kyoto (WKY) rat after injection of sodium nitroprusside (SNP). **B**, Representative trace showing MAP, BP, and HR in a WKY rat after injection of phenylephrine. **C**, Representative trace showing MAP, BP, and HR in an SHR after injection of SNP. **D**, Representative trace showing MAP, BP, and HR in an SHR after injection of phenylephrine. **E**, Group data showing BP and HR before injections (baseline) in SHRs (red) and WKY rats (black). **F**, Group data showing BP and HR after injections of phenylephrine (Pe) and SNP in SHRs (red) and WKY rats (black). Data are represented as mean±SD with individual data points.

Our SHRs were hypertensive (WKY: 97±14.1 MAP, n=4 versus SHR: 188.2±16.1 MAP, n=3; *P*=0.001; Figure 1E) but did not display altered heart rate (WKY: 128.8±18.4 beats per minute [BPM], n=3 versus SHR: 176.1±66.3 BPM, n=5; Figure 1E). Therefore, their hypertension is uncompensated, indicating that they have been hypertensive for a long period.^23^

Following phenylephrine injection, SHRs have increased blood pressure (phenylephrine: WKY: 57.4±6.2 MAP, n=4 versus SHR: 82.8±2.3 MAP, n=3; *P*=0.002; Figure 1A, 1B, and 1F) and heart rate (phenylephrine: WKY: 59.9±4.8 BPM, n=3 versus SHR: 82.3±5.6 BPM, n=4; *P*=0.003; Figure 1A, 1B, and 1F), demonstrating increased sensitivity to vasoconstriction. Injection of sodium nitroprusside did not affect the blood pressure (WKY: 186.6±52.8 MAP, n=5 versus SHR: 215.3±73.7 MAP, n=5; Figure 1C, 1D, and 1F) or heart rate (WKY: 199.9±54.4 BPM, n=4 versus SHR: 208.2±65.8 BPM, n=5; Figure 1C, 1D, and 1F), likely because vasodilation is impaired to counter chronic high blood pressure in prolonged hypertension.^24^

### SHRs Have Innate SA Without Alterations in Respiration During Wakefulness

**Figure 2.**
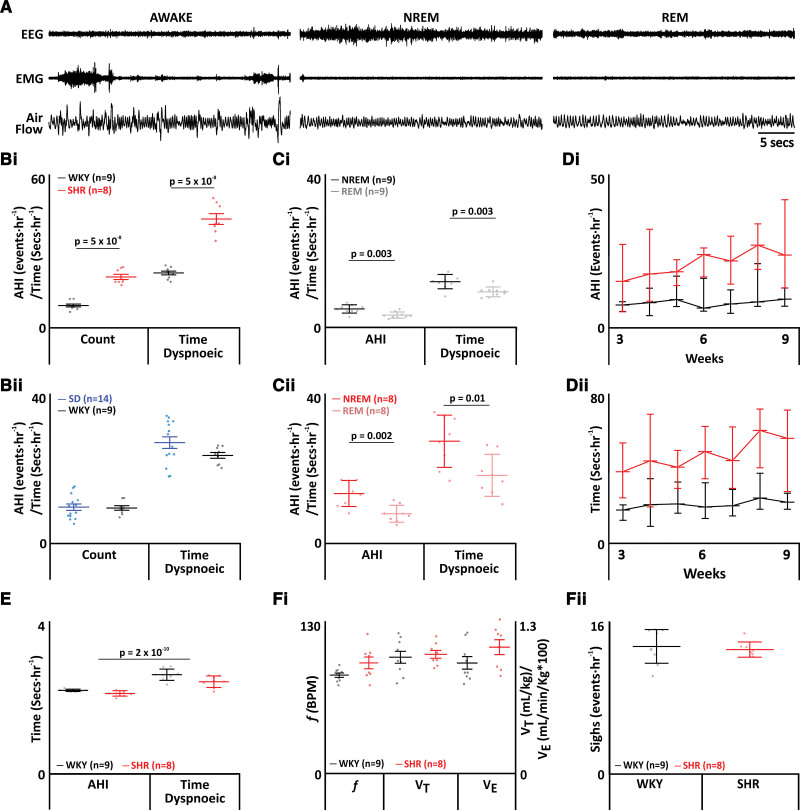
**Spontaneously hypertensive rats (SHRs) display apneas with no change in breathing. A**, Plethysmograph traces from SHRs, showing respiratory movements as measured by changes in airflow in a plethysmograph, with electroencephalographic (EEG) and electromyographic (EMG) electrodes for assigning sleep-wake state. (**B**) through (**F**) Data represent Wistar-Kyoto (WKY) rats (black) compared with SHRs (red). **B**, Frequency of breathing disturbances (apnea-hypopnea index [AHI]) and duration spent dyspneic (time) between WKY rats and SHRs (**Bi**) and between SDs (blue) and WKY rats (**Bii**). **C**, Frequency of breathing disturbances and duration spent dyspneic by sleep state, nonrapid eye movement (NREM; black), and rapid eye movement (REM; gray) in WKY rats (**Ci**) and in NREM (red) and REM (pink) in SHRs (**Cii**). **D**, Progression of AHI (**Di**) and time spent dyspneic (**Dii**) by week. **E**, Average duration of dyspnea during NREM and REM. **F**, Respiratory parameters: during wakefulness (**Fi**). Left axis shows values for frequency (f) and right axis for tidal volume (V_T_) and minute ventilation (V_e_). **Fii**, Frequency of sighing. Data are represented as mean±SD with individual data points.

To determine whether SHRs displayed innate SA, we measured their breathing and sleep-wake state (Figure 2A). Apnea-hypopnea index (sum of apneic+hypopneic events per hour of sleep) was increased in SHRs (WKY: 9±0.6 incidences per hour of sleep, n=9 versus SHR: 20.7±1.1 incidences per hour of sleep^1^, n=6; *P*=0.00000005; Figure 2Bi) as was total duration of apneas+hypopneas (WKY: 22.4±0.8 s, n=9 versus SHR: 44.5±2.2 s, n=8; *P*=0.00000005; Figure 2Bi). SA in WKY rats was no different to Sprague-Dawley controls^17^ for apnea-hypopnea index (Sprague-Dawley: 9.4±0.8 incidences per hour of sleep, n=14 versus WKY: 9.2±0.6 incidences per hour of sleep, n=9; Figure 2Bii) or total time spent dyspneic (Sprague-Dawley: 26.2±1.5 s, n=14 versus WKY: 22.9±0.8 s, n=9; Figure 2Bii).

For both WKY rats and SHRs, apneas occurred more in nonrapid eye movement (NREM) sleep than in rapid eye movement (REM) sleep (WKY: NREM: 5.4±0.4 incidences per hour of sleep, n=9 versus REM: 3.8±0.4 incidences per hour of sleep, n=9; *P*=0.003; SHR: NREM: 12.8±1.1 incidences per hour of sleep, n=8 versus REM: 7.9±0.8 incidences per hour of sleep, n=8; *P*=0.002; Figure 2Ci and 2Cii). There was also an increase in time spent dyspneic during NREM (WKY: NREM: 12.7±2 s, n=9 versus REM: 9.9±0.4 s, n=9; *P*=0.003; SHR: NREM: 26.3±2.3 s, n=8 versus REM: 18.2±2 s, n=8; *P*=0.01; Figure 2Ci and 2Cii).

As time progressed, there was no difference seen in the number of dyspneic episodes or duration of the dyspneic events (Figure 2Di and 2Dii) and no difference in the average duration of dyspneic events (WKY: 2.4±0.2 s, n=9 versus SHR: 2.3±0.2 s, n=8; Figure 2E).

There was no difference in respiratory parameters during wakefulness, SHR maintained respiratory frequency *f* (WKY: 87±15 breaths/min, n=9 versus SHR: 98±6 breaths/min, n=8; Figure 2Fi), tidal volume (WKY: 1.0±0.1 mL/kg, n=9 versus SHR: 1.0±0.0 mL/kg, n=9; Figure 2Fi), and minute ventilation (WKY: 89±5 mL/kg/min, n=9 versus SHR: 101±6 mL/kg/min, n=8; Figure 2Fi). There was no difference in sigh rate (WKY: 13±1.8 incidences per hour, n=9 versus SHR: 13±0.8 incidences per hour, n=8; Figure 2Fii); thus, the overall integrity of the respiratory microcircuit remained intact.

### SHRs Have Reduced Neurons in the preBötC

**Figure 3.**
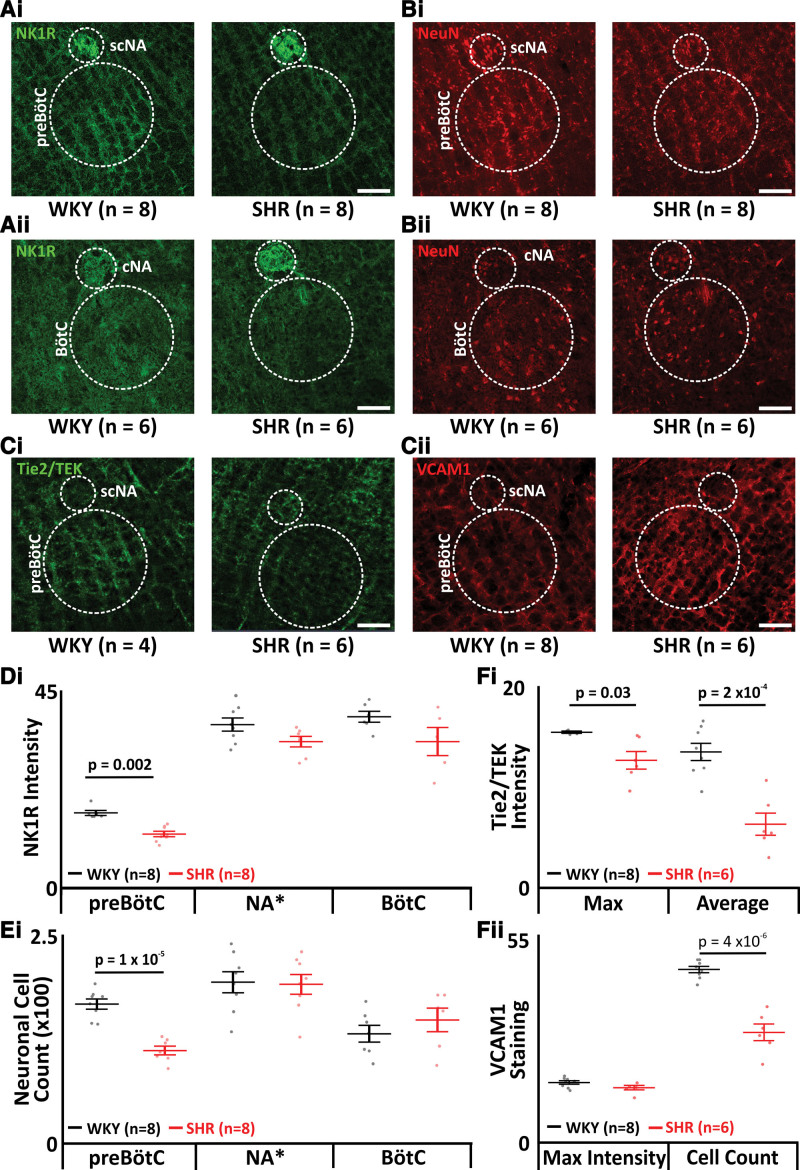
**Spontaneously hypertensive rats (SHRs) display a neuronal and vascular deficit in the predefined area of the preBötC. A**, Micrographs showing immunocytochemical staining of NK1R (neurokinin-1 receptor) positive cells (green) of the preBötzinger Complex (preBötC) and semicompact nucleus ambiguus (scNA; **Ai**), and Bötzinger Complex (BötC) and compact nucleus ambiguus (cNA; **Aii**). **B**, Micrographs showing immunocytochemical staining of the neurons (NeuN [neuronal nuclear antigen], red) of the preBötC and scNA (**Bi**) and the BötC and cNA (**Bii**). **C**, Micrographs showing immunocytochemical staining of vasculature in the preBötC and scNA through Tie2/TEK (angiopoietin-1 receptor) positive cells (green; **Ci**) and VCAM1 (vascular cell adhesion molecule 1) positive cells (VCAM1: red; **Cii**). **D**, Group data of the NK1R stain intensities in the preBötC nucleus ambiguus (NA) and BötC shown in **A. E**, Group data of the neuronal cell counts of the preBötC, NA and BötC shown in **B. F**, Group data of the vascular staining in the preBötC. Tie2/TEK maximum and average intensity staining intensity are represented by the micrographs shown in **Ci**. Group data of the maximum intensity and cell counts of VCAM1 preBötC staining intensity are represented by the micrographs shown in **Cii**. Data are represented as mean±SD with individual data points. WKY indicates Wistar-Kyoto.

To assess whether the SA in SHRs is due to loss of preBötC neurons, we analyzed the preBötC, Bötzinger Complex, and nucleus ambiguus. SHRs have a loss of presumptive rhythm-generating cells in the preBötC (WKY: 29.6±7.0 NK1R intensity, n=8 versus SHR: 16.5±4.1 NK1R intensity, n=8; *P*=0.002; Figure 3Ai and 3D) with no difference in the nucleus ambiguus (WKY: 37.5±4.3 NK1R intensity, n=8 versus SHR: 35.0±4.8 NK1R intensity, n=8; Figure 3Ai and 3D) or Bötzinger Complex (WKY: 18.0±0.9 NK1R intensity, n=7 versus SHR: 17.4±0.7 NK1R intensity, n=6; Figure 3Aii and 3D). There was a general loss of preBötC neurons (WKY: 1745±179 neurons, n=8 versus SHR: 1164±143 neurons, n=7; *P*=0.000001; Figure 3Bi and 3E) with no loss of nucleus ambiguus (WKY: 202±37 neurons, n=8 versus SHR: 199±35 neurons, n=7; Figure 3Bi and 3E) or Bötzinger complex neurons (WKY: 1374±105 neurons, n=7 versus SHR: 1547±148 neurons, n=6; Figure 3Bii and 3E). A loss of 33% of preBötC exceeds the threshold required to induce SA, explaining why SHRs are afflicted by it.

To understand why preBötC neurons were dying, we investigated vascular health. We found a loss of vasculature in the preBötC, measured by Tie2/TEK average intensity (WKY: 13.5±2.4 intensity, n=8 versus SHR: 6.3±2.7 intensity, n=8; *P*=0.0002; Figure 3Ci and 3Fi) and maximum intensity (WKY: 15.6±0.5 intensity, n=6 versus SHR: 12.6±0.9 intensity, n=6; *P*=0.03; Figure 3Ci and 3Fi). There was a reduction in VCAM1 positive cells (WKY: 47.3±2.5 cells, n=8 versus SHR: 29.8±5.6 cells, n=6; *P*=0.000004; Figure 3Cii and 3Fii). VCAM1 maximal intensity remained unchanged (WKY: 15.9±0.5 intensity, n=8 versus SHR: 14.5±0.6 intensity, n=6; Figure 3Cii and 3Fii). Given that the vasculature showed the same level of damage (unchanged VCAM1 maximal intensity), the reduction in VCAM1 positive cells is due to the loss of vasculature (decreased Tie2/TEK staining).

### SHRs Display No Change in Time Spent in REM

**Figure 4.**
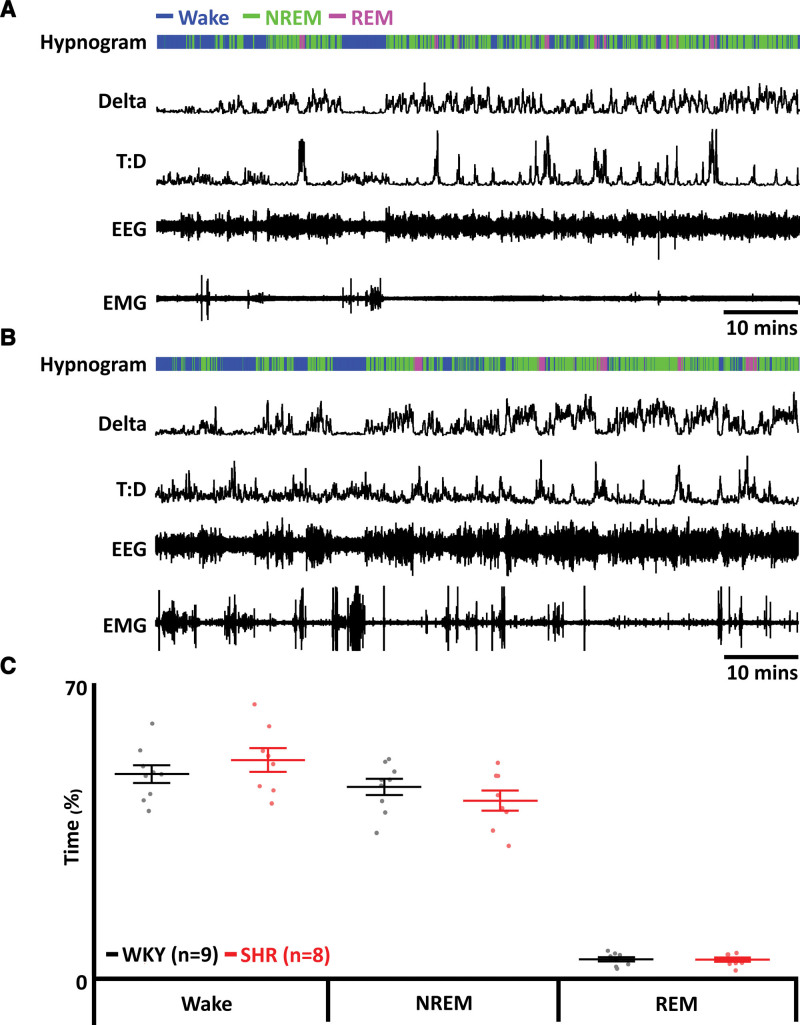
**Spontaneously hypertensive rats (SHRs) display no change in time spent in rapid eye movement (REM). A**, Data from a representative Wistar-Kyoto (WKY) rat. **B**, Data from a representative SHR rat. **A** and **B**, Electromyographic (EMG) recordings were used to determine activity and to identify periods of REM and nonrapid eye movement (NREM) sleep. Electroencephalographic (EEG) recordings were used to determine delta wave activity for NREM sleep and to calculate theta-delta ratio (T:D) for REM sleep. Hypnograms show time spent awake and in REM and NREM sleep stages. **C**, Time spent in wakefulness and NREM and REM sleep in SHRs (red) compared with WKY rats (black). Data are represented as mean±SD with individual data points.

Dyspneas are observed during both phases of sleep and induce microarousals (Figure 4A and 4B). There were no sleep disturbances seen in either group for either sleep state (wake: WKY: 49±6%, n=9 versus SHR: 52±8%, n=8; Figure 4C; NREM: WKY: 46±6%, n=9 versus SHR: 42±7%, n=8; Figure 4C; and REM: WKY: 5±1%, n=9 versus SHR: 5±1%, n=8; Figure 4C). While we did not see altered sleep, sleep fragmentation in SHRs occurs at a higher frequency at the end of the light phase.^25^ Therefore, the lack of sleep fragmentation is because we only captured a snapshot of the animal’s sleep that did not always span this period.

### SHRs Have Neuroinflammation

**Figure 5.**
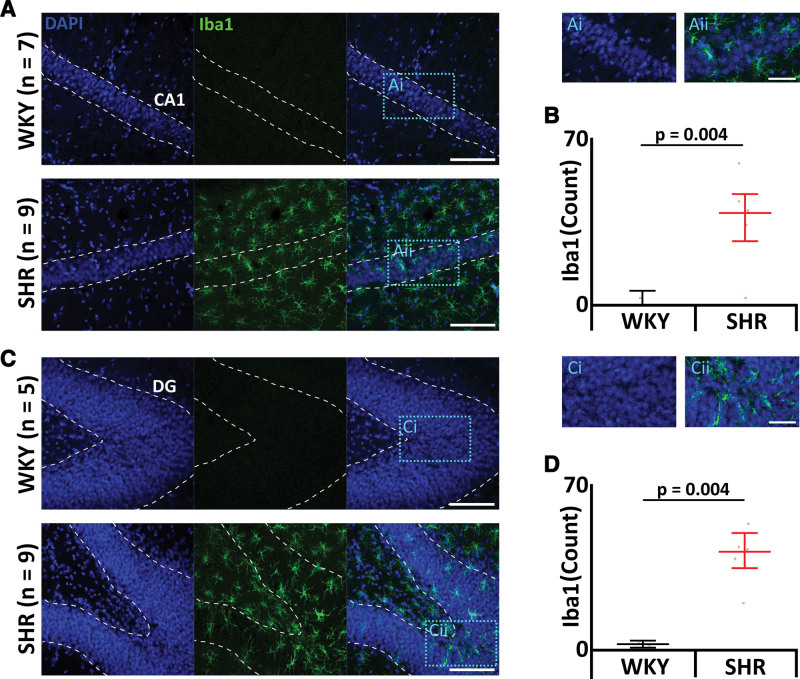
**Spontaneously hypertensive rats (SHRs) display elevated levels of activated microglia. A** and **C**, Micrographs showing activated microglia, a marker of neural inflammation, through Iba1(ionized calcium-binding adaptor molecule 1) expression. Scale bars: 100 µm. Blue boxes represent expanded areas shown in **i** and **ii**. Expanded micrograph scale bars: 50 µm. **A**, CA1 (cornu ammonis-1) region. **C**, Dentate gyrus (DG). **B** and **D**, Group data displayed in (**B**) CA1 region and (**D**) DG. Data are represented as mean±SD with individual data points. WKY indicates Wistar-Kyoto.

Central SA (CSA) induced through cytotoxic loss of preBötC neurons leads to neuroinflammation in the hippocampus, which is associated with decreased cognitive flexibility in both long and short memory.^17^ The reduction in preBötC neurons, and levels of SA, in SHRs is comparable to our surgical model of moderate SA.^17^ Given the parallels, we next investigated whether SHRs showed neuroinflammation in the hippocampus. SHRs showed activated microglia in the hippocampus in both the CA1 (cornu ammonis-1) region (WKY: 4±6 cells, n=6 versus SHR: 36±16 cells, n=9; *P*=0.004; Figure 5A and 5B) and dentate gyrus (WKY: 5±6 cells, n=5 versus SHR: 40±10 cells, n=9; *P*=0.004; Figure 5C and 5D).

### SHRs Have Deficits in Long-Term Memory

**Figure 6.**
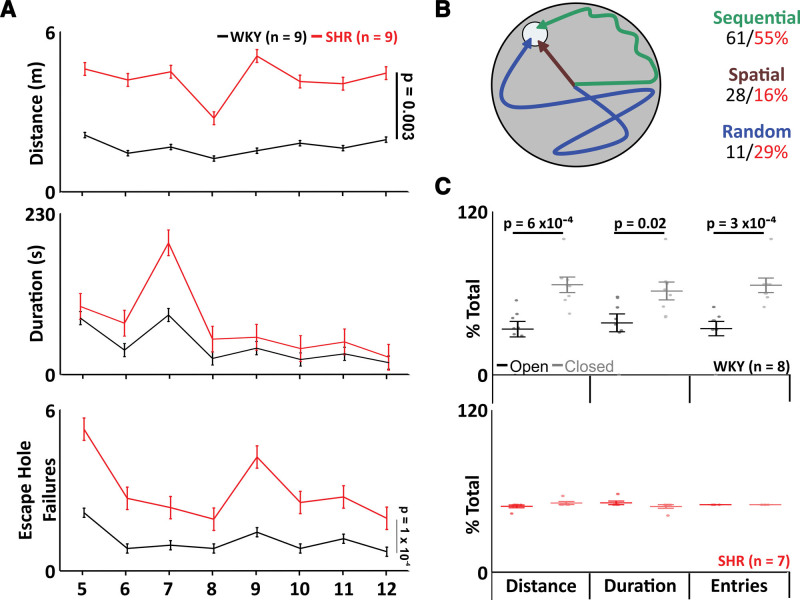
**Spontaneously hypertensive rats (SHRs) display short- and long-term memory declines.** A and B, Barnes maze. **A**, Group data showing time spent and distance covered on the maze, as well as the number of escape hole failures in SHRs (red) compared with Wistar-Kyoto (WKY) rats (black). **B**, Cartoon displays search strategies used by SHRs (red) compared with WKY rats (black). **C**, Group data showing the number of entries, distance covered, and time spent in the novel arm (light shading) compared with the previously open arm (dark shading) in SHRs (red) compared with WKY rats (black). Data are represented as mean±SD with individual data points.

SHRs spent a similar amount of time on the Barnes maze (WKY: 39±10 s versus SHR: 33±11 s; mean difference, 6; SE, 15; DF, 14; Figure 6A) but cover more distance (WKY: 1.7±0.1 m versus SHR: 2.5±0.2 m; mean difference, –8; SE, 2.2; DF, 14; *P*=0.003; Figure 6A) and have more escape hole failures (WKY: 0.7±0.1 versus SHR: 1.7±0.1; mean difference, –0.2; SE, 0.2; DF, 14; *P*=0.00001; Figure 6A). Therefore, SHRs have altered long-term memory.

There was no difference in search strategies between WKY and SHR (spatial WKY: 28±6% versus SHR: 16±3%; sequential WKY: 61±8% versus SHR: 55±6%; random WKY: 11±5% versus SHR: 29±5%; Figure 6B).

### SHRs Have Deficits in Short-Term Memory

In a Y maze forced alteration test, SHRs did not display a preference for the novel arm for distance traveled (WKY: open, 34±7%; closed, 66±7%; *P*=0.0006; n=8; and SHR: open, 52±1%; closed, 48±1%; n=7; Figure 6C), duration (WKY: open, 39±7%; closed, 61±7%; *P*=0.02; n=8; SHR: open, 54±1%; closed, 4±1%; n=7; Figure 6C), or entries (WKY: open, 34±6%; closed, 66±6%; *P*=0.0003; n=8; SHR: open, 52±0%; closed, 48±0%; n=7; Figure 6C).

Both groups covered the same total distance (WKY: 8.9±2.2 m, n=6 versus SHR: 9±2.2 m, n=7; data not shown), implicating that locomotive and anxiety were unaffected in SHRs.

## DISCUSSION

Hypertension, the silent killer, causes blood vessel damage and vascular remodeling, affecting all major organs including the brain. Over time, blood vessels thicken, reducing elasticity, narrowing the vessel, and increasing vascular resistance.^26^ This combination of atherosclerosis/arteriolosclerosis and hypertension causes microvascular rarefaction,^27^ the loss of small blood vessels and microcirculation, and worsening the symptoms of hypertension.^28^ This reduction in microvascular density is caused either by reduced angiogenesis (more commonly in genetic hypertension) or vessel destruction (more commonly in secondary hypertension). In the brain, this causes cerebral small vessel disease, which can progress to cognitive decline.^29^

Interestingly, the majority of NREM-related apneas that we saw were terminated by arousal, indicative of obstructive SA.^30^ The recurrent laryngeal nerve can be damaged by hypertension-dependent aldosterone release causing inflammation-induced edema of the airways.^31^ This nerve innervates the larynx, controlling basic functions such as talking, swallowing, and breathing.^32^ Damaging this nerve reduces muscle tone, allowing the tongue to obstruct airflow during sleep, resulting in apnea.^33^

We found evidence of microvascular rarefaction in the preBötC in SHRs, via reduced intensity of Tie2/TEK, a tyrosine kinase receptor central to vascular stability.^34^ This receptor has 3 ligands (Ang1, Ang2, and Ang4), each angiopoietin.^35^ Tie2/TEK signaling complements the vascular endothelial growth factor pathway impacting vascular development.^35^ We postulate that the density of microvasculature in the preBötC has been reduced through diminished angiogenesis over a prolonged period. Tie2 activation upregulates VCAM1,^36^ which, in turn, upregulates transmigration of leukocytes across membranes.^37^ A loss of VCAM1 is indicative of overall loss of vascular tissue, most likely due to inappropriate activation of the cell adhesion molecule enhancing chronic inflammation^38^; here, in the preBötC. Interestingly, while we saw a 39% decrease in NK1R positive neurons and a 33% decrease in preBötC neurons overall, we saw no loss of neurons in the Bötzinger complex and no change in the number of sighs per hour of sleep, which suggests that the damage caused by microvascular rarefaction is differentially regulated throughout the respiratory column. Whether this selective response is regulated through differential sensitivity to oxidative stress, hypertension, or other factors remains unknown.

Inhibitory preBötC neurons (both inspiratory and expiratory) modulate cardiac parasympathetic neuronal activity,^39^ whereas excitatory preBötC neurons modulate sympathetic vasomotor neuronal activity.^40^ Loss of neurons in the preBötC has profound effects on cardiovascular output, including respiratory sinus arrhythmia^41^ and exacerbation of hypertension.^40^ Loss of both excitatory and inhibitory neurons, therefore, would lead to impaired respiratory entrainment, with exaggerated effects on blood pressure and heart rate in hypertension. We predict that the long-term effects of the lack of entrainment would exacerbate the microvascular effects of preexisting hypertension.

CSA, a lack of ventilatory drive and respiratory effort during sleep, differs significantly from obstructive SA, where respiratory effort is observed during a dyspneic episode.^42^ REM-related dyspneic episodes were not terminated by arousal, and the arousal instead follows a period of hyperpnea to reoxygenate tissue, indicative of CSA.^43^ The pathophysiology of CSA may contribute to the instability of respiration during sleep by causing chronic inflammation, as intermittent hypoxia can inhibit phrenic long-term facilitation.^44,45^

Characteristics from both obstructive SA and CSA are present in SHRs, presenting as a mixed model of SA: with predicted damage to the recurrent laryngeal nerve by prolonged hypertension causing obstructive SA, and loss of preBötC neurons caused by microvascular rarefaction inducing CSA. SA worsens atherosclerosis/arteriolosclerosis in the brain through the production of NOS (NO synthase)^46^ disrupting the vascular tone of arteries,^47^ resulting in a loss of brain volume. Interestingly, where there is a decrease in upregulation of endothelial NOS below its optimum limit, there is less regulation of blood pressure, less angiogenesis, and inappropriate vascular remodeling,^48^ as well as the direct recruitment of activated microglia and upregulation of proinflammatory cytokines in the brain.^49^

We saw increased microglial activation in both the CA1 and dentate gyrus regions of the hippocampus, which are associated with memory consolidation,^50–52^ as well as impaired long- and short-term memories in SHRs. Prolonged microglial activation is responsible for increased damage to the blood-brain barrier increasing permeability, causing leukocyte recruitment to the brain.^53^ Some classes of leukocytes depend on VCAM1 for their migration across endothelia into the brain.^54^ Once within the tissue, leukocytes are acted upon by natural T killer cells, regulating their performance as reparative leukocytes. However, in some cases, for example, during sterile inflammation induced by SA,^17^ this switch does not occur and leukocytes initiate inappropriate constant cycles of damage and repair of tissue as they are unable to transmigrate back into the bloodstream,^55^ resulting in chronic inflammation as the cycle perpetuates. Inflammation in the brain results in white matter injury, primarily as a result of widespread demyelination,^56^ which may be accompanied by significant gray matter loss.^57^ Myelin is particularly susceptible to death as a result of inflammation, and without the protective myelin sheath, the white matter also becomes more at risk of death provoking loss of volume.^58^

We conclude that the cognitive decline present in SHRs was exacerbated due to increased neuroinflammation, which may increase microvascular rarefaction induced by prolonged hypertension^59^ and chronic intermittent hypoxia.^60^

## ARTICLE INFORMATION

### Acknowledgments

R. Roberts performed experiments and analyzed data. Dr Huckstepp designed and performed experiments, analyzed data, and oversaw the project. Both authors wrote the article and approved the submitted version. The authors thank Prof Johannes Boltze for his valuable input and conversations about the spontaneously hypertensive rat phenotype.

### Sources of Funding

This work was supported by the University of Warwick, Medical and Life Sciences Research Fund Bursary and the Biotechnology and Biological Sciences Research Council (grant number: BB/X008290/1).

### Disclosures

None.

### Supplemental Material

Expanded Materials and Methods

Figure S1

## Supplementary Material



## References

[1] Lim LL Tham KW Fook-Chong SM. Obstructive sleep apnoea in Singapore: polysomnography data from a tertiary sleep disorders unit. Ann Acad Med Singap. 2008;37:629–636. 18797554

[2] Heinzer R Vat S Marques-Vidal P Marti-Soler H Andries D Tobback N Mooser V Preisig M Malhotra A Waeber G. Prevalence of sleep-disordered breathing in the general population: the HypnoLaus study. Lancet Respir Med. 2015;3:310–318. doi: 10.1016/S2213-2600(15)00043-0 25682233 10.1016/S2213-2600(15)00043-0 PMC4404207

[3] Franklin KA Lindberg E. Obstructive sleep apnea is a common disorder in the population-a review on the epidemiology of sleep apnea. J Thorac Dis. 2015;7:1311–1322. doi: 10.3978/j.issn.2072-1439.2015.06.11 26380759 10.3978/j.issn.2072-1439.2015.06.11 PMC4561280

[4] Franklin KA Sahlin C Stenlund H Lindberg E. Sleep apnoea is a common occurrence in females. Eur Respir J. 2013;41:610–615. doi: 10.1183/09031936.00212711 22903961 10.1183/09031936.00212711

[5] Zhou B Carrillo-Larco RM Danaei G Riley LM Paciorek CJ Stevens GA Gregg EW Bennett JE Solomon B Singleton RK. Worldwide trends in hypertension prevalence and progress in treatment and control from 1990 to 2019: a pooled analysis of 1201 population-representative studies with 104 million participants. Lancet. 2021;398:957–980. doi: 10.1016/S0140-6736(21)01330-1 34450083 10.1016/S0140-6736(21)01330-1 PMC8446938

[6] Peppard PE Young T Palta M Skatrud J. Prospective study of the association between sleep-disordered breathing and hypertension. New Eng J Med. 2000;342:1378–1384. doi: 10.1056/nejm200005113421901 10805822 10.1056/NEJM200005113421901

[7] Young T Peppard P Palta M Hla KM Finn L Morgan B Skatrud J. Population-based study of sleep-disordered breathing as a risk factor for hypertension. Arch Intern Med. 1997;157:1746–1752. doi: 10.1001/archinte.1997.00440360178019 9250236

[8] Haas DC Foster GL Nieto FJ Redline S Resnick HE Robbins JA Young T Pickering TG. Age-dependent associations between sleep-disordered breathing and hypertension. Circulation. 2005;111:614–621. doi: 10.1161/01.CIR.0000154540.62381.CF 15699282 10.1161/01.CIR.0000154540.62381.CF

[9] Wang M Brage S Sharp SJ Luo S Au Yeung SL Kim Y. Associations of genetic susceptibility and healthy lifestyle with incidence of coronary heart disease and stroke in individuals with hypertension. Eur J Prev Cardiol. 2022;29:2101–2110. doi: 10.1093/eurjpc/zwac135 35788660 10.1093/eurjpc/zwac135

[10] Sierra C. Hypertension and the risk of dementia. Front Cardiovasc Med. 2020;7:5. doi: 10.3389/fcvm.2020.00005 32083095 10.3389/fcvm.2020.00005 PMC7005583

[11] Culebras A Anwar S. Sleep apnea is a risk factor for stroke and vascular dementia. Curr Neurol Neurosci Rep. 2018;18:53. doi: 10.1007/s11910-018-0855-1 29938308 10.1007/s11910-018-0855-1

[12] Peracaula M Torres D Poyatos P Luque N Rojas E Obrador A Orriols R Tura-Ceide O. Endothelial dysfunction and cardiovascular risk in obstructive sleep apnea: a review article. Life (Basel). 2022;12:537. doi: 10.3390/life12040537 35455027 10.3390/life12040537 PMC9025914

[13] Rundek T Tolea M Ariko T Fagerli EA Camargo CJ. Vascular cognitive impairment (VCI). Neurotherapeutics. 2022;19:68–88. doi: 10.1007/s13311-021-01170-y 10.1007/s13311-021-01170-y PMC9130444 34939171

[14] Whitmer RA Sidney S Selby J Johnston SC Yaffe K. Midlife cardiovascular risk factors and risk of dementia in late life. Neurology. 2005;64:277–281. doi: 10.1212/01.WNL.0000149519.47454.F2 15668425 10.1212/01.WNL.0000149519.47454.F2

[15] Smith JC Ellenberger HH Ballanyi K Richter DW Feldman JL. Pre-Bötzinger complex: a brainstem region that may generate respiratory rhythm in mammals. Science. 1991;254:726–729. doi: 10.1126/science.1683005 1683005 10.1126/science.1683005 PMC3209964

[16] McKay LC Feldman JL. Unilateral ablation of pre-Bötzinger complex disrupts breathing during sleep but not wakefulness. Am J Respir Crit Care Med. 2008;178:89–95. doi: 10.1164/rccm.200712-1901OC 18420958 10.1164/rccm.200712-1901OC PMC2441928

[17] Roberts R Wall MJ Braren I Dhillon K Evans A Dunne J Nyakupinda S Huckstepp RTR. An improved model of moderate sleep apnoea for investigating its effect as a comorbidity on neurodegenerative disease. Front Aging Neurosci. 2022;14:861344. doi: 10.3389/fnagi.2022.861344 35847678 10.3389/fnagi.2022.861344 PMC9278434

[18] Kaiser D Weise G Möller K Scheibe J Pösel C Baasch S Gawlitza M Lobsien D Diederich K Minnerup J. Spontaneous white matter damage, cognitive decline and neuroinflammation in middle-aged hypertensive rats: an animal model of early-stage cerebral small vessel disease. Acta Neuropathol Commun. 2014;2:169. doi: 10.1186/s40478-014-0169-8 25519173 10.1186/s40478-014-0169-8 PMC4279586

[19] Suvila K Langén V Cheng S Niiranen TJ. Age of hypertension onset: overview of research and how to apply in practice. Curr Hypertens Rep. 2020;22:68. doi: 10.1007/s11906-020-01071-z 32852608 10.1007/s11906-020-01071-z PMC7452883

[20] Carley DW Berecek K Videnovic A Radulovacki M. Sleep-disordered respiration in phenotypically normotensive, genetically hypertensive rats. Am J Respir Crit Care Med. 2000;162:1474–1479. doi: 10.1164/ajrccm.162.4.9911033 11029364 10.1164/ajrccm.162.4.9911033

[21] Mayor AH Schwartz AR Rowley JA Willey SJ Gillespie MB Smith PL Robotham JL. Effect of blood pressure changes on air flow dynamics in the upper airway of the decerebrate cat. Anesthesiology. 1996;84:128–134. doi: 10.1097/00000542-199601000-00015 8572325 10.1097/00000542-199601000-00015

[22] Percie du Sert N Ahluwalia A Alam S Avey MT Baker M Browne WJ Clark A Cuthill IC Dirnagl U Emerson M. Reporting animal research: explanation and elaboration for the ARRIVE guidelines 20. PLoS Biol. 2020;18:e3000411. doi: 10.1371/journal.pbio.3000411 32663221 10.1371/journal.pbio.3000411 PMC7360025

[23] Reule S Drawz PE. Heart rate and blood pressure: any possible implications for management of hypertension? Curr Hypertens Rep. 2012;14:478–484. doi: 10.1007/s11906-012-0306-3 22972532 10.1007/s11906-012-0306-3 PMC3491126

[24] Lind L Granstam SO Millgård J. Endothelium-dependent vasodilation in hypertension: a review. Blood Press. 2000;9:4–15. doi: 10.1080/080370500439362 10854002

[25] Lai CT Chen CY Kuo TBJ Chern CM Yang CCH. Sympathetic hyperactivity, sleep fragmentation, and wake-related blood pressure surge during late-light sleep in spontaneously hypertensive rats. Am J Hypertens. 2016;29:590–597. doi: 10.1093/ajh/hpv154 26350298 10.1093/ajh/hpv154

[26] Lusis AJ. Atherosclerosis. Nature. 2000;407:233–241. doi: 10.1038/35025203 11001066 10.1038/35025203 PMC2826222

[27] Austin TR Nasrallah IM Erus G Desiderio LM Chen LY Greenland P Harding BN Hughes TM Jensen PN Longstreth WT Jr. Association of brain volumes and white matter injury with race, ethnicity, and cardiovascular risk factors: the multi-ethnic study of atherosclerosis. J Am Heart Assoc. 2022;11:e023159. doi: 10.1161/JAHA.121.023159 35352569 10.1161/JAHA.121.023159 PMC9075451

[28] Kerkhove D Paciolla I Arpino G. Chapter 3 - classification by mechanisms of cardiotoxicity. In: Lancellotti P Zamorano Gómez JL Galderisi M, eds. Anti-Cancer Treatments and Cardiotoxicity. Academic Press; 2017:13–34.

[29] Yulu S Joanna MW. Update on cerebral small vessel disease: a dynamic whole-brain disease. Stroke Vasc Neurol. 2016;1:83. doi: 10.1136/svn-2016-000035 28959468 10.1136/svn-2016-000035 PMC5435198

[30] Dingli K Fietze I Assimakopoulos T Quispe-Bravo S Witt C Douglas NJ. Arousability in sleep apnoea/hypopnoea syndrome patients. Eur Respir J. 2002;20:733–740. doi: 10.1183/09031936.02.00262002 12358354 10.1183/09031936.02.00262002

[31] Bangash A Wajid F Poolacherla R Mim FK Rutkofsky IH. Obstructive sleep apnea and hypertension: a review of the relationship and pathogenic association. Cureus. 2020;12:e8241. doi: 10.7759/cureus.8241 32582500 10.7759/cureus.8241 PMC7306640

[32] Culp JM Patel G. Recurrent Laryngeal Nerve Injury. StatPearls. 2023. 32809667

[33] Novakovic D MacKay S. Adult obstructive sleep apnoea and the larynx. Curr Opin Otolaryngol Head Neck Surg. 2015;23:464–469. doi: 10.1097/MOO.0000000000000209 26488535 10.1097/MOO.0000000000000209

[34] Gál Z Gézsi A Molnár V Nagy A Kiss A Sultész M Csoma Z Tamási L Gálffy G Bálint BL. Investigation of the possible role of tie2 pathway and TEK gene in asthma and allergic conjunctivitis. Front Genet. 2020;11:702. doi: 10.3389/fgene.2020.00702 32754197 10.3389/fgene.2020.00702 PMC7381303

[35] Augustin HG Koh GY Thurston G Alitalo K. Control of vascular morphogenesis and homeostasis through the angiopoietin-Tie system. Nat Rev Mol Cell Biol. 2009;10:165–177. doi: 10.1038/nrm2639 19234476 10.1038/nrm2639

[36] Anisimov A Fang S Hemanthakumar KA Örd T van Avondt K Chevre R Toropainen A Singha P Gilani H Nguyen SD. The angiopoietin receptor Tie2 is atheroprotective in arterial endothelium. Nat Cardiovasc Res. 2023;2:307–321. doi: 10.1038/s44161-023-00224-y 37476204 10.1038/s44161-023-00224-y PMC7614785

[37] Kong DH Kim YK Kim MR Jang JH Lee S. Emerging roles of vascular cell adhesion molecule-1 (VCAM-1) in immunological disorders and cancer. Int J Mol Sci. 2018;19:1057. doi: 10.3390/ijms19041057 29614819 10.3390/ijms19041057 PMC5979609

[38] Cook-Mills JM Marchese ME Abdala-Valencia H. Vascular cell adhesion molecule-1 expression and signaling during disease: regulation by reactive oxygen species and antioxidants. Antioxid Redox Signal. 2011;15:1607–1638. doi: 10.1089/ars.2010.3522 21050132 10.1089/ars.2010.3522 PMC3151426

[39] Cui Y Kam K Sherman D Janczewski WA Zheng Y Feldman JL. Defining preBötzinger complex rhythm- and pattern-generating neural microcircuits in vivo. Neuron. 2016;91:602–614. doi: 10.1016/j.neuron.2016.07.003 27497222 10.1016/j.neuron.2016.07.003 PMC4978183

[40] Menuet C Connelly AA Bassi JK Melo MR Le S Kamar J Kumar NN McDougall SJ McMullan S Allen AM. PreBötzinger complex neurons drive respiratory modulation of blood pressure and heart rate. Elife. 2020;9:e57288. doi: 10.7554/eLife.57288 32538785 10.7554/eLife.57288 PMC7326498

[41] Furuya WI Dhingra RR Trevizan-Baú P McAllen RM Dutschmann M. The role of glycinergic inhibition in respiratory pattern formation and cardio-respiratory coupling in rats. Curr Res Physiol. 2021;4:80–93. doi: 10.1016/j.crphys.2021.03.001 34746829 10.1016/j.crphys.2021.03.001 PMC8562146

[42] Eckert DJ Jordan AS Merchia P Malhotra A. Central sleep apnea: pathophysiology and treatment. Chest. 2007;131:595–607. doi: 10.1378/chest.06.2287 17296668 10.1378/chest.06.2287 PMC2287191

[43] Hanly BJ Millar TW Steljes DG Boert R Frais MA Kryger MH. Respiration and abnormal sleep in patients with congestive heart failure. Chest. 1989;96:480–488. doi: 10.1378/chest.96.3.480 2766808 10.1378/chest.96.3.480

[44] Hocker AD Stokes JA Powell FL Huxtable AG. The impact of inflammation on respiratory plasticity. Exp Neurol. 2017;287:243–253. doi: 10.1016/j.expneurol.2016.07.022 27476100 10.1016/j.expneurol.2016.07.022 PMC5121034

[45] Devinney MJ Huxtable AG Nichols NL Mitchell GS. Hypoxia-induced phrenic long-term facilitation: emergent properties. Ann NY Acad Sci. 2013;1279:143–153. doi: 10.1111/nyas.12085 23531012 10.1111/nyas.12085 PMC3880582

[46] Valko M Leibfritz D Moncol J Cronin MTD Mazur M Telser J. Free radicals and antioxidants in normal physiological functions and human disease. Int J Biochem Cell Biol. 2007;39:44–84. doi: 10.1016/j.biocel.2006.07.001 16978905 10.1016/j.biocel.2006.07.001

[47] Matthys KE Bult H. Nitric oxide function in atherosclerosis. Mediators Inflamm. 1997;6:3–21. doi: 10.1080/09629359791875 18472828 10.1080/09629359791875 PMC2365844

[48] An L Shen Y Chopp M Zacharek A Venkat P Chen Z Li W Qian Y Landschoot-Ward J Chen J. Deficiency of endothelial nitric oxide synthase (eNOS) exacerbates brain damage and cognitive deficit in a mouse model of vascular dementia. Aging Dis. 2021;12:732–746. doi: 10.14336/AD.2020.0523 34094639 10.14336/AD.2020.0523 PMC8139201

[49] Katusic ZS Austin SA. Endothelial nitric oxide: protector of a healthy mind. Eur Heart J. 2014;35:888–894. doi: 10.1093/eurheartj/eht544 24357508 10.1093/eurheartj/eht544 PMC3977136

[50] Hainmueller T Bartos M. Dentate gyrus circuits for encoding, retrieval and discrimination of episodic memories. Nat Rev Neurosci. 2020;21:153–168. doi: 10.1038/s41583-019-0260-z 32042144 10.1038/s41583-019-0260-z PMC7115869

[51] Bahar AS Shirvalkar PR Shapiro ML. Memory-guided learning: CA1 and CA3 neuronal ensembles differentially encode the commonalities and differences between situations. J Neurosci. 2011;31:12270–12281. doi: 10.1523/JNEUROSCI.1671-11.2011 21865470 10.1523/JNEUROSCI.1671-11.2011 PMC3167378

[52] Minhas PS Latif-Hernandez A McReynolds MR Durairaj AS Wang Q Rubin A Joshi AU He JQ Gauba E Liu L. Restoring metabolism of myeloid cells reverses cognitive decline in ageing. Nature. 2021;590:122–128. doi: 10.1038/s41586-020-03160-0 33473210 10.1038/s41586-020-03160-0 PMC8274816

[53] Zhou H Lapointe BM Clark SR Zbytnuik L Kubes P. A requirement for microglial TLR4 in leukocyte recruitment into brain in response to lipopolysaccharide. J Immunol. 2006;177:8103–8110. doi: 10.4049/jimmunol.177.11.8103 17114485 10.4049/jimmunol.177.11.8103

[54] Leick M Azcutia V Newton G Luscinskas FW. Leukocyte recruitment in inflammation: basic concepts and new mechanistic insights based on new models and microscopic imaging technologies. Cell Tissue Res. 2014;355:647–656. doi: 10.1007/s00441-014-1809-9 24562377 10.1007/s00441-014-1809-9 PMC3994997

[55] Zindel J Kubes P. DAMPs, PAMPs, and LAMPs in immunity and sterile inflammation. Annu Rev Pathol: Mech Dis. 2020;15:493–518. doi: 10.1146/annurev-pathmechdis-012419-032847 10.1146/annurev-pathmechdis-012419-032847 31675482

[56] Rossi S Motta C Studer V Macchiarulo G Volpe E Barbieri F Ruocco G Buttari F Finardi A Mancino R. Interleukin-1β causes excitotoxic neurodegeneration and multiple sclerosis disease progression by activating the apoptotic protein p53. Mol Neurodegener. 2014;9:56. doi: 10.1186/1750-1326-9-56 25495224 10.1186/1750-1326-9-56 PMC4292815

[57] Trapp BD Vignos M Dudman J Chang A Fisher E Staugaitis SM Battapady H Mork S Ontaneda D Jones SE. Cortical neuronal densities and cerebral white matter demyelination in multiple sclerosis: a retrospective study. Lancet Neurol. 2018;17:870–884. doi: 10.1016/S1474-4422(18)30245-X 30143361 10.1016/S1474-4422(18)30245-X PMC6197820

[58] Souza-Rodrigues RD Costa AMR Lima RR Dos Santos CD Picanço-Diniz CW Gomes-Leal W. Inflammatory response and white matter damage after microinjections of endothelin-1 into the rat striatum. Brain Res. 2008;1200:78–88. doi: 10.1016/j.brainres.2007.11.025 18289508 10.1016/j.brainres.2007.11.025

[59] Antonios TF. Microvascular rarefaction in hypertension—reversal or over-correction by treatment? Am J Hypertens. 2006;19:484–485. doi: 10.1016/j.amjhyper.2005.11.010 16647619 10.1016/j.amjhyper.2005.11.010

[60] Nanduri J Peng YJ Yuan G Kumar GK Prabhakar NR. Hypoxia-inducible factors and hypertension: lessons from sleep apnea syndrome. J Mol Med (Berl). 2015;93:473–480. doi: 10.1007/s00109-015-1274-2 25772710 10.1007/s00109-015-1274-2 PMC4409567

