# Supplementary material for: Innate Sleep Apnea in Spontaneously Hypertensive Rats Is Associated With Microvascular Rarefaction and Neuronal Loss in the preBötzinger Complex
Source: Stroke. 2023 Nov 28;54(12):3141–52. doi: 10.1161/STROKEAHA.123.044732 (PMC10769171; doi:10.1161/STROKEAHA.123.044732)
Supplement: Supplementary file 1 [file str-54-3141-s001.pdf]

SUPPLEMENTAL MATERIAL

Surgeries

Adult (6-8 months old) male Wistar Kyoto rats (WKY: 417 ± 35g, *n* = 9) and Spontaneously Hypertensive rats (SHR: 406 ± 54g, *n* = 8). The order of the rats pseudorandomized prior to surgery. Five animals were excluded as they did not complete the study (in total 22 rats were used in the study). Initial group sizes were determined through power analysis on AHI (see plethysmography section below) performed in GPower3.1.

Rats were induced and maintained on 0.5-2% inhalation Isoflurane (Piramal Healthcare, Mumbai, India) in pure oxygen (1 L·min^{-1}) throughout the surgery. Rats were administered sub-cutaneous Meloxicam (2 mg·kg^{-1}; Norbrook Inc., Lenexa, KA, United States) and Atropine (0.12 mg·kg^{-1}; Westward Pharmaceutical co., Tujunga, CA United States) to provide long-term moderate analgesia and to prevent pleural effusion, respectively. Post-operatively rats are given Buprenorphine (0.1 mg·kg^{-1}; Reckitt Benckiser, Slough, United Kingdom) to provide additional short-term analgesia. Rats were placed prone in a stereotaxic apparatus (Kopf Instruments, Tujunga, CA, United States) thermocoupled to a heating pad (TCAT 2-LV; Physitemp, Clifton, NJ, United States) and body temperature was maintained at a minimum of 33°C. Anaesthetic level was monitored throughout the surgery.

EEG/EMG

For all animals, four electroencephalographic (EEG) electrodes were inserted into the brain, two cross-cortical electrodes which predominantly measure delta waves (**Figure S1A**; electrodes 1 and 2), and 2 cross-hippocampus electrodes which predominantly measure theta waves (**Figure S1A**; electrodes 3 and 4). Two electromyographic (EMG) wires were inserted into the trapezius muscle to record REM induced paralysis. The EEG electrodes (M1.4 x 3mm Philips Pan Head Machine Screws (DIN 7985H) – Stainless Steel (A2), SIP – M1.4-3-A2; Accu, Huddersfield, United Kingdom) and EMG wire are connected to a custom-built head mount fixed in place with Superbond dental cement (Prestige Dental, Bedworth, United Kingdom) and Vertex Orthoplast cold-curing orthodontic acrylic resin (Prestige Dental, Bedworth, United Kingdom). Animals were allowed to recover 2 weeks post-operative before recordings began, with food and water *ad libitum,* at ambient room temperature, 22 ± 2°C. Rats were pseudorandomised before every phase of testing and following the surgery the experimenters were blinded to the condition of each rat.

Plethysmography

Rats were placed in to a 4.5 L plethysmography chamber (**Figure S1B**) with airflow set at 2 L·min^{-1} and calibrated via a 1 mL syringe, with an average ambient chamber temperature of 23 ± 0.9°C: the rats showed no signs of thermoregulation (panting, shivering, excessive locomotion, abnormal sleeping patterns). During the second week post-surgery, rats were allowed two acclimation sessions; once the plethysmograph was placed inside the rat's home cage for 30 min, and on a separate occasion rats were placed into the plethysmograph for 30 min under experimental conditions. After these acclimation sessions, each rat underwent one 3 h recording per week for 7 weeks (postsurgical weeks 3-9 inclusive) during the light phase of the 12 h Light/dark cycle.

Airflow was recorded via a pressure transducer connected to the plethysmography chamber. Tidal volume (V_T) was measured from trough-to-peak of each breath. Frequency (*f*) of breathing is measured from peak-peak of inspiration and is given as breaths·min^{-1}. Minute ventilation (Ve) is determined by multiplying V_T x *f*. The respiratory parameters were measured during quiet wakefulness at the beginning of the recording. The signals from the pressure transducer were filtered and amplified using the Neurolog system via a 1401 interface (Digitimer, Welwyn Garden City, United Kingdom), and all data was acquired with Spike2 software (Cambridge Electronic Design, Cambridge, United Kingdom).

For assessment of the severity of SA, we measured the length of the respiratory disturbances (apnoea-hypoxia Index (AHI: sum of apnoeic + hypopnoeic events per hour of sleep)) by hand. Apnoea is defined as decrease in minute ventilation of 90% or greater for 1.8 s or more, hypopnoea is defined as decrease in minute ventilation of 50-90% for 1.8 s or more. Sighs (a compound breath followed by a period of apnoea) per hour of quiet wake + sleep were determined using EMG and flow traces; when the EMG was low with little/no movement shown combined with a clean period on the breathing trace this was considered quiet wake or sleep.

Sleep-Wake Recordings

Theta waves were recorded from electrodes 1 (placed in the dorsal hippocampus) and 2 (placed in the frontal cortex) (**Figure S1A**), and delta waves were recorded from electrodes 3 and 4 (**Figure S1A**) then the EEG signals were amplified 7,500-fold and bandpass filtered at 5-70 Hz. EMG signals were recorded through the wire in the trapezoid muscle and amplified 4,500-fold and bandpass filtered at 50-1,500 Hz. All data was processed with the Spike2 software using the OSD script, and sleep scoring was aided with video footage from an HD camera of the rat throughout the entire 3 hour recording where physiological factors such as locomotion (WAKE), or paralysis (REM) are used to aid with analysis of sleep-wake state. EEG signals that had been band-pass filtered with a power spectrum between 0-4 Hz were classed as delta waves, and signals between 6-10 Hz were classed as theta waves. All EEG signals were smoothed with a 5 s time constant after being band-pass filtered. The EMG signals were then processed with a 5 s RMS constant, and the whole 3 h recording is separated into 5 s epochs.

Combining EEG and EMG analysis allowed us to determine sleep wake state into 4 distinct categories:

WAKE: low amplitude desynchronised EEG, high amplitude and frequency on EMG

NREM: high amplitude EEG delta waves, low amplitude and frequency on EMG

REM: low amplitude EEG theta waves and high theta:delta (T:D) ratio, low amplitude (or absent) EMG

DOUBT: any epoch that cannot be defined into one of the aforementioned states

All sleep state and AHI count analysis are expressed as per hour of sleep for consistency across the recordings, and sigh counts are expressed as per combined hour of sleep and quiet wakefulness.

Cognitive Tests

Y Maze (forced alternation)

The Y maze (**Figure S1A**) was adapted from a radial arm maze displayed so that 3 arms (50 X 10 cm with 13 cm high walls) in the shape of the letter Y remained open (Stoelting, Dublin, Ireland). This phase of behavioural testing takes place either before or after the Barne's maze in post-surgical week 10 or 13, the determination of which is pseudorandomised at the start of the experimental paradigm. The rat is placed into the maze in the entry arm, and either the left or the right arm is open, with the remaining arm closed. The location of the closed arm is pseudorandomised before each test. Spatial cues are placed at the end of each arm in plain sight of the rat, with comfortable ambient lighting (150-250 lux). Rats were given 10 mins to explore the maze, rested for a period of 1 h before being retested for 5 mins with both arms open. The order of the rats is pseudorandomised before the first test but is consistent between the tests. All test sessions are recorded with a camera (Henelec Model 335 BWL; Sony, Surrey, United Kingdom) that connects to a computer for offline analysis (Any-MAZE v4.96, Stoelting, Dublin, Ireland). Total time spent in each arm, total distance travelled in each arm and the total number of entries into each arm (defined as 20% of the rat's whole body in the arm) were measured.

Barnes Maze

The Barnes maze is a circular maze (122 cm in diameter) with 19 false holes and one escape hole (9 cm in diameter; Stoelting, Dublin, Ireland) (**Figure S1D**). The maze is one metre off the ground. Spatial cues of different colours and shapes are spaced evenly around the maze at regular intervals in plain sight of the rat. Spatial clues are never placed directly over the escape hole. The surface of the table is brightly lit (>1500 lux) to create an adverse environment. On post-surgical week 10, rats underwent a 12-day Barnes maze protocol: Days 1-3 (learning phase) the escape hole contains an incentive (peanut butter); days 4-12 (acquisition phase) the incentive is provided in the home cage after the test, allowing the rat to be rewarded for completing the test but removing the olfactory stimulus from the maze. Before each test the order of the rats is pseudorandomised. The maze is thoroughly cleaned (70% ethanol) between tests to remove any traces of scent from the previous rat. No respiratory or other behavioural experiment occur on the days where the Barnes maze is performed to remove the impact these may have on the cognitive testing. The experiment is recorded by a camera (Henelec Model 335 BWL; Sony, Surrey, United Kingdom) connected to a computer for offline analysis (Any-MAZE v4.96, Stoelting, Dublin, Ireland). Exit errors (defined as the total number of times the rat investigated the escape hole but did not exit through it), total distance travelled on the maze and total time spent on the maze is measured. Search strategy was divided into 3 specific categories; sequential (travelling around the outside of the maze visiting ≥ 3 holes), spatial (travelling directly to within 2 holes of the escape hole and not visiting any other hole outside of this quadrant) or random (no specific pattern but must cross the centre line ≥ 2 times).

Baroreflex Sensitivity Test

Rats were anaesthetised in a gas inhalation chamber using 4% isoflurane (Piramal Healthcare, Mumbai, India) in pure oxygen and maintained throughout surgery with urethane (1 g/kg; Sigma: St. Louis, Missouri, United States) and α-chloralose (50 $mg\cdot kg^{-1}$;Sigma: St. Louis, Missouri, United States) diluted in standard sterile saline (0.9% NaCl). Additional doses of α-chloralose were administered as required. Anaesthetic levels are checked throughout with pedal withdrawal, and body temperature was maintained at 36.5°C by a thermocoupled heat mat. The trachea was cannulated. The femoral blood vessels were catheterised: the femoral vein was used for delivery of anaesthetics and the femoral artery was connected to pressure transducers (Digitimer, Welwyn Garden City, United Kingdom) to record blood pressure (**Figure S1E**).

Baseline parameters were recorded for 20 mins. Phenylephrine (3.5 $mg\cdot mL^{-1}$; Fisher Scientific UK, Loughborough, Leicestershire, LE11 5Rg, United Kingdom) or sodium nitroprusside (35 $mg\cdot mL^{-1}$; Honeywell, Chem-Supply Pty Ltd, 38-50 Bedford Street, Gillman SA 5013, Australia) were administered followed by a saline flush. The animal was allowed to rest until measurements returned to baseline, or a new baseline was established over 20 mins before administration of the second drug. The order of the drugs was pseudorandomised before the surgery starts. Mean arterial pressure (MAP) was calculated as [⅔ Diastolic pressure + ⅓ Systolic Pressure] and heart rate (BPM) was calculated as peak to peak of blood pressure and is given as $beats\cdot min^{-1}$. Data are displayed as the ratios of the MAP or heart rate after injection compared to the average baseline of each animal.

Tissue Collection

At the end of the baroreflex-sensitivity tests rats were transcardially perfused with 4% paraformaldehyde (PFA). The cortex and the medulla were removed and fixed overnight in 4% PFA at 4°C. Once the brains had sunk (typically 2-3 days) they were transferred to, and stored in, cryoprotectant (30% sucrose + 0.02% sodium azide) at 4°C.

Immunocytochemistry

Tissue was sectioned to 50 µm on a cryostat (Bright instruments Ltd, Huntington, United Kingdom). Slices were washed in PBS for six x 5-min washes, before being placed in a sodium citrate buffer (tri-sodium citrate (dihydrate) 2.94g, 1 L ddH_2O, 0.5 mL Tween 20, adjusted to pH 9.0 with NaOH), preheated to 80°C in a still water bath for (heat-induced) antigen retrieval. The free-floating slices were left for 30 minutes, before being washed in PBS for 6 x 5-minute washes. Tissue was transferred to blocking solution (PBS containing 0.1% Triton X and 5% bovine-serum albumin) to block non-specific binding of antibodies for 1 h at room temperature. The slices were then incubated overnight at room temperature in the blocking solution plus the following primary antibodies:

*Medulla (**Figure S1F**)*

preBötC counts: mouse anti-NeuN (1:100, MAB337, Merck Millipore, Watford, United Kingdom), rabbit anti-NK1R (1:500, ab5060, Merck Millipore, Watford, United Kingdom).

Microvascular rarefaction: rabbit anti-VCAM (1:200, ab134047, Abcam, Cambridge, United Kingdom), mouse anti-Tie2/TEK (1:200, ab33, Merck Millipore, Watford, United Kingdom), goat anti-ChAT (1:50, ab144P, Merck Millipore, Watford, United Kingdom).

Hippocampus

Neuroinflammation: goat anti-Iba-1 (1:83, ab5076, Abcam, Cambridge, United Kingdom).

The slices were then washed in PBS for six x 5-minute washes, then placed in blocking solution for 1 h at room temperature. The slices were then incubated for 2 h at room temperature with the following secondary antibodies:

Medulla

preBötC counts: donkey anti-mouse Alexa Fluor 568 (1:250, A10037, Invitrogen, Waltham, MA, United States), donkey anti-rabbit Alexa Fluor 488 (1:250, A10037, Invitrogen, Waltham, MA, United States).

Microvascular rarefaction: donkey anti-rabbit Alexa Fluor 568 (1:250, ab150074, Abcam, Cambridge, United Kingdom), donkey anti-mouse Alexa Fluor 488 (1:250, A-21202,Hampton, Fisher Scientific, New Hampshire, United States), donkey anti-goat Alexa Fluor 405 (1:250, ab175665, Abcam, Cambridge, United Kingdom);

Hippocampus

Neuroinflammation: donkey anti-goat Alexa Fluor 488 (1:250, A10037, Invitrogen, Waltham, MA, United States), DAPI staining solution (1:1000, ab228549, Abcam, Cambridge, United Kingdom).

All slices were then mounted on polylysine microscope slides and dehydrated overnight at room temperature. The slides were then rehydrated in ddH_2O, mounted (Cytoseal 60, Electron Microscopy Sciences, Hatfield, PA, United States) and coverslipped.

Slides were imaged on a confocal microscope (Zeiss 880, Zeiss, Jena, Germany) using Zen Blue and Zen Black software (Zeiss, Jena, Germany).

Medulla analysis: The number of NeuN-positive cells were counted to determine neuronal loss within a 600 µm diameter circle below the semi-compact nucleus ambiguous, defining the preBötC. Following a background subtraction on the staining for either NK1R-positive, VCAM-positive or Tie2/TEK-positive neurons, fluorescence intensity was measured within the preBötC. The same intensity analysis and

cell count analysis was performed on the nucleus ambiguus for the NK1R and NeuN analysis to confirm the continuity of staining across all tissue.

Hippocampus analysis: the dentate gyrus or the CA1 region were imaged. To identify activated microglia, we counted DAPI-stained nuclei encapsulated with Iba1 anywhere in the visual field. Given we only stained every second slice, nuclei could only be present in a single section, and thus removing overestimation by double counting.

All image processing and analysis was performed on ImageJ (ImageJ, U.S. National Institutes of Health, Bethesda, MA, United States).

Experimental Design

For each stage of the experimental timeline (**Figure S1G)**, the order of the rats was pseudorandomised, and for the duration of the experimental timeline, the experimenter(s) were blinded to the condition (hypertensive vs normotensive). The plethysmograph acclimation occurred in the 2nd week post-surgery, and the experimental plethysmography recordings started in the 3rd week post-surgery and took place weekly up to and inclusive of the 9th week post-surgery. Rats then either underwent a 1-day Y maze protocol or a 12-day Barnes maze protocol, the order of which was pseudorandomised for each batch of rats. The other maze was then completed after a 3-day rest period. One week after the final maze session, the baroreflex sensitivity experiments were performed, and tissue was collected, and. Once all experiments were finished and all analysis was completed, then the experimenters were unblinded.

Data analysis

All experimental units are a rat, and all technical repeats are averaged to create a biological repeat for analysis. An Iglewicz & Hoaglin's robust test (with a modified Z score of ≥ 3.5 htpps://contchart.com/outliers.aspx) was used to remove outliers before statistical analyses were carried out. To establish the state of normality, Shapiro-Wilks tests were carried out on the sham-operated groups. All experiments are recorded and analysed using Spike software (vs7.08, Cambridge Electronic Design).

Data for baseline MAP ($p = 0.9$), Pe MAP ($p = 0.3$), SNP MAP ($p = 0.2$), baseline HR ($p = 0.2$), Pe HR ($p = 0.8$), SNP HR ($p = 0.6$), preBötC NeuN cell count ($p = 0.5$), Tie2/TEK preBötC staining intensity ($p = 0.8$), VCAM1 preBötC staining intensity ($p = 0.1$), preBötC VCAM1 cell counts ($p = 0.6$), BötC staining intensity ($p = 0.2$), BötC cell count ($p = 0.9$), NA NK1R staining intensity ($p = 0.9$), NA cell count ($p = 0.9$), V_T ($p = 0.7$), V_E ($p = 0.1$), f ($p = 0.9$), average sighs ($p = 0.2$), number of dyspnoeic episodes during sleep ($p = 0.9$), time spent dyspnoeic ($p = 0.3$), time spent in disordered breathing by sleep state (WKY: $p = 0.9$; SHR: $p = 0.9$), Wake ($p = 0.8$), NREM ($p = 0.5$), REM ($p = 0.8$) were deemed Gaussian by a Shapiro-Wilks test for normality and tested by a two sample t test. Data are expressed as mean ± SD.

Data for preBötC NK1R staining intensity ($p = 0.003$), CA1 Iba1 positive cell count ($p = 0.05$), DG Iba1 positive cell count ($p = 0.00007$), Barnes maze search strategies ($p = 0.003$) were deemed non-Gaussian by a Shapiro-Wilks test for normality and tested by a Kruskal Wallis ANOVA with Dunn-Sidak post-hoc correction. Data are expressed as mean ± SD.

Data for average length of dyspnoeic episode ($p = 0.4$) was deemed Gaussian by a Shapiro-Wilks test for normality and tested by a two-way ANOVA with Bonferroni correction. Data are expressed as mean ± SD.

Data for Y Maze entries (p = 0.008) was deemed non-Gaussian by a Shapiro-Wilks test for normality and tested by a two-way repeated measures ANOVA with Bonferroni correction. Data are expressed as mean ± SD.

Data for number of dyspnoeic episodes by week (p = 0.3) and time spent dyspnoeic by week (p = 0.5), Y Maze duration (p = 0.9), Y Maze distance (p = 0.8), Y Maze discrimination ratio (distance (p = 0.5), duration (p = 0.2), entries (p = 0.2)), were deemed Gaussian by a Shapiro-Wilks test for normality and tested by a two-way repeated measures ANOVA with Bonferroni correction. Data are expressed as mean ± SD.

Data for Barnes maze duration (p = 0.4) was deemed non-Gaussian, Barnes maze distance (p = 0.1), and Barnes maze exit errors (p = 0.1) were deemed Gaussian by a Shapiro-Wilks test for normality and tested by a two-way repeated measures ANOVA with a Dunn-Sidak correction. Data are expressed as mean ± SD.

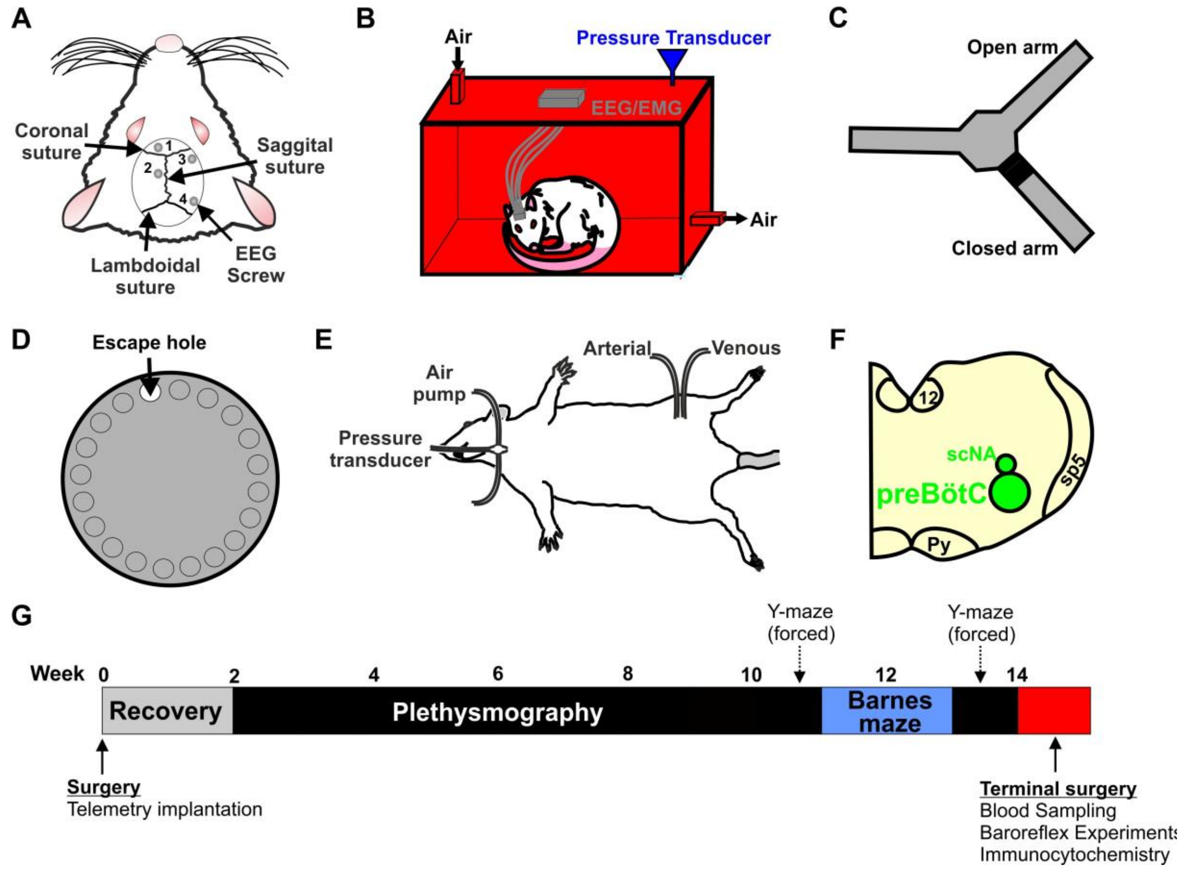

Figure 1 **|** *Testing innate sleep apnoea (SA) in SHR.* **(A)** Electroencephalographic (EEG) and Electromyographic (EMG) electrodes were used to measure sleep-wake states in Spontaneously Hypertensive Rats (SHR) and Wistar Kyoto Rats (WKY). **(B)** A custom-built 4.5L plethysmograph was used to house the rats during the 3 h recording where EEG/EMG signals and flow (via a pressure transducer) were recorded. **(C)** Short-term memory was measured using a Y-Maze (forced alteration). **(D)** Long-term memory was measured using a Barnes Maze over a 12-day protocol. **(E)** Blood pressure, heart rate and breathing frequency recordings were taken whilst the rat was maintained under terminal anaesthesia and oxygen. **(F)** Immunocytochemical staining of the pre-defined area of the preBötzinger Complex (preBötC) and semi-compact Nucleus Ambiguus (scNA) was performed at the end of the experimental timeline. **(G)** Experimental timeline.
